# First-Trimester Hepatobiliary and Inflammatory Composite Indices as Candidate Markers for Subsequent Intrahepatic Cholestasis of Pregnancy: A Case–Control Study

**DOI:** 10.3390/diagnostics16172745

**Published:** 2026-08-27

**Authors:** Hanne Bulat Cim, Nisan Helin Dönmez, Melis Altuğ Inan, Canan Satır Ozel, Ergül Demirçivi, Neslişah Ar, Abdulkadir Turgut

**Affiliations:** 1 Department of Obstetrics and Gynecology, Göztepe Prof. Dr. Süleyman Yalçın City Hospital, Istanbul 34722, Türkiye; nisanuzulmez@hotmail.com (N.H.D.); melaltug@gmail.com (M.A.I.); 2 Department of Obstetrics and Gynecology, School of Medicine, Istanbul Medeniyet University, Göztepe Prof. Dr. Süleyman Yalçın City Hospital, Istanbul 34722, Türkiye; drcanansatirozel@hotmail.com (C.S.O.); drergul@yahoo.com (E.D.); neslisahar99@gmail.com (N.A.); abdulkadirturgut@gmail.com (A.T.)

**Keywords:** intrahepatic cholestasis of pregnancy, first trimester, S-index, De Ritis ratio, hepatobiliary biomarkers, risk stratification, inflammatory indices

## Abstract

**Background/Objectives:** Intrahepatic cholestasis of pregnancy (ICP) typically becomes apparent in the second or third trimester, although hepatobiliary susceptibility may be present earlier. We evaluated associations between first-trimester hepatobiliary and inflammatory composite indices and subsequent ICP. **Methods:** This retrospective case–control study included 96 women who developed ICP and 197 controls. The S-index, De Ritis ratio, albumin–bilirubin score (ALBI), gamma-glutamyl transferase-to-platelet ratio (GPR), transaminase complex-to-platelet ratio (TACPR), neutrophil-to-lymphocyte ratio (NLR), platelet-to-lymphocyte ratio (PLR), monocyte-to-lymphocyte ratio (MLR), and systemic inflammation response index (SIRI) were calculated from measurements from 8 + 0 to 13 + 6 weeks of gestation. ROC analysis, DeLong comparisons, and multivariable logistic regression were performed. **Results:** Hepatobiliary indices showed numerically higher AUCs than inflammatory indices. The S-index had the numerically highest observed AUC (0.808; 95% CI, 0.727–0.889), with 66.7% sensitivity and 95.1% specificity at a cutoff of ≥0.045. The De Ritis ratio had an AUC of 0.717. DeLong comparisons showed that the S-index had a significantly higher AUC than ALBI but did not differ significantly from the De Ritis ratio, GPR or TACPR. After adjustment for age, body mass index and gestational age at blood sampling, the S-index and De Ritis ratio remained associated with ICP. No inflammatory index had an AUC significantly greater than 0.50. **Conclusions:** First-trimester hepatobiliary indices, including the S-index and De Ritis ratio, were associated with subsequent ICP and may reflect an early hepatobiliary pattern. These indices warrant prospective validation as adjunctive markers for ICP risk stratification and for identifying women who may benefit from closer surveillance and timely or repeated bile acid testing.

## 1. Introduction

Intrahepatic cholestasis of pregnancy (ICP) is a pregnancy-specific hepatobiliary disorder that typically presents during the second or third trimester with pruritus in the absence of a primary skin eruption and elevated serum total bile acid concentrations [1]. Although maternal symptoms generally resolve after delivery, ICP is associated with clinically important perinatal complications, including spontaneous and iatrogenic preterm birth, meconium-stained amniotic fluid, neonatal morbidity, and stillbirth [2]. Serum total bile acid concentrations remain central to both diagnosis and fetal risk assessment, and increasing concentrations are associated with a progressively greater risk of adverse perinatal outcomes [3]. The pathogenesis of ICP is multifactorial, involving genetic susceptibility, pregnancy-related hormonal influences, and impaired canalicular bile acid transport, which together promote maternal bile acid accumulation and potential fetal and placental toxicity [4].

Although ICP typically becomes clinically apparent later in pregnancy, the biological susceptibility underlying the disorder may be present considerably earlier. Genetic variants affecting hepatobiliary transport pathways are present before pregnancy, whereas increasing concentrations of estrogen and sulfated progesterone metabolites during gestation may progressively challenge a limited capacity to maintain normal bile acid homeostasis [5,6,7]. Within this framework, subtle alterations in routine liver-related laboratory parameters during the first trimester may represent an early biochemical imprint of limited hepatobiliary adaptability rather than clinically established cholestasis. Previous studies have suggested that changes in biochemical, platelet, or inflammatory parameters may be detectable before the clinical diagnosis of ICP, supporting further investigation of routinely available first-trimester markers [8,9,10]. If prospectively validated, first-trimester markers associated with subsequent ICP could help identify women who may benefit from closer surveillance for characteristic symptoms and timely or repeated bile acid testing when clinically indicated. This may facilitate the timely initiation of symptom-directed treatment, antenatal surveillance, and bile acid-guided obstetric management after diagnosis [1].

Composite indices may improve the detection of such subtle changes by integrating complementary biochemical and hematological parameters into a single measure. Previous studies in ICP have evaluated the De Ritis ratio, GPR, ALBI, TACPR, and several complete blood count-derived inflammatory indices; however, most have focused on individual markers, limited groups of indices, or measurements obtained at or near the time of diagnosis [10,11,12,13,14]. The S-index, originally developed as a GGT-, platelet-, and albumin-based score for chronic liver disease, has been less extensively evaluated in ICP, whereas TACPR has only recently been proposed as a transaminase- and platelet-based composite marker [12,15]. Accordingly, a gap appears to remain in the literature regarding the simultaneous first-trimester comparison of these hepatobiliary and inflammatory indices within the same cohort, particularly with respect to the evaluation of the less extensively studied S-index and the recently proposed TACPR alongside established indices.

The present study aimed to compare the discriminative performance of nine routinely calculable first-trimester hepatobiliary and inflammatory composite indices for subsequent ICP within the same cohort. We also assessed which marker class demonstrated greater discrimination and whether the leading candidate indices remained associated with ICP in multivariable models. By directly comparing these two marker classes, we sought to identify routinely available candidate markers that could inform the future development and external validation of first-trimester risk-stratification models for ICP.

## 2. Materials and Methods

### 2.1. Study Design, Setting, and Ethical Approval

This single-center retrospective case–control study was conducted at the Department of Obstetrics and Gynecology, Göztepe Prof. Dr. Süleyman Yalçın City Hospital, Istanbul, Türkiye. The electronic medical records of pregnant women managed between January 2015 and January 2025 were reviewed. The study protocol was approved by the Ethics Committee of Göztepe Prof. Dr. Süleyman Yalçın City Hospital (approval no. 2025/0303; 20 November 2025) and was conducted in accordance with the principles of the Declaration of Helsinki. The requirement for informed consent was waived by the ethics committee because of the retrospective design of the study.

### 2.2. Study Population and Eligibility Criteria

Pregnant women were eligible if they were 18–45 years of age, had a singleton pregnancy, received antenatal care from the first trimester onward, and had complete first-trimester laboratory data for all components required to calculate the investigated indices. First-trimester measurements were defined as those obtained between 8 + 0 and 13 + 6 weeks of gestation and were required to precede the diagnosis of ICP.

The fasting serum total bile acid measurements used for group classification were obtained during the second or third trimester. The ICP group comprised women with otherwise unexplained pruritus accompanied by a fasting serum total bile acid concentration >10 µmol/L, after the exclusion of alternative hepatic, biliary, dermatologic, and pregnancy-related causes. The control group was derived from the same clinical population and study period and comprised women who underwent fasting serum total bile acid testing because of clinical suspicion of ICP but were not diagnosed with ICP through delivery and had fasting total bile acid concentrations <10 µmol/L at all available measurements.

Women were excluded if they had chronic liver disease, including viral hepatitis, cirrhosis, autoimmune hepatitis, or primary biliary cholangitis; a history of alcohol-related liver disease or excessive alcohol consumption; or exposure to medications known to substantially affect hepatic function. Additional exclusion criteria were pregestational diabetes mellitus, chronic hypertension, chronic kidney disease, autoimmune or hematologic disorders, multiple pregnancy, and other pregnancy-related liver disorders, including preeclampsia, HELLP syndrome, and acute fatty liver of pregnancy. Women with active infection, systemic inflammatory disease, antibiotic treatment, or hospitalization for an acute condition at the time of first-trimester blood sampling were also excluded. Records lacking any laboratory component required for calculation of the investigated indices were excluded from the analytical cohort.

### 2.3. Data Sources and Laboratory Measurements

Demographic, clinical, laboratory and delivery data were obtained from the hospital electronic medical record and laboratory information systems. Maternal age and BMI were recorded at the first antenatal assessment during the first trimester as part of routine antenatal care. The laboratory panel obtained closest to the end of the predefined first-trimester window of 8 + 0 to 13 + 6 weeks of gestation was used for analysis. When more than one measurement was available during this period, the most recent assessment was selected to capture the biochemical profile at the end of the first trimester. All components required for the calculation of a given index were obtained from the same laboratory assessment to ensure temporal comparability.

Routine serum biochemical and complete blood count measurements were performed in the hospital’s central laboratory using automated analyzers and standardized laboratory procedures in use at the time of testing. The current institutional laboratory reference range defines the upper limit of normal for GGT as 40 U/L; this value was applied uniformly to all participants for the calculation of GPR. Umbilical cord blood gas analysis was not routinely performed in all newborns during the study period; therefore, cord blood pH was available only for neonates in whom it had been obtained as part of routine clinical care.

### 2.4. Calculation of Composite Indices

The hepatobiliary composite indices were calculated from first-trimester laboratory parameters as follows: De Ritis ratio = AST/ALT; ALBI = 0.66 × log10[total bilirubin (µmol/L)] − 0.085 × albumin (g/L); GPR = {[(GGT [U/L]/upper limit of normal for GGT [U/L]) × 100]}/platelet count (×10^9^/L); TACPR = [AST (U/L) × ALT (U/L)]/platelet count (×10^9^/L); and S-index = [1000 × GGT (U/L)]/{platelet count (×10^9^/L) × [albumin (g/L)]^2^}.

The inflammatory indices were calculated from first-trimester complete blood count parameters as follows: systemic inflammation response index (SIRI) = (neutrophil count × monocyte count)/lymphocyte count; platelet-to-lymphocyte ratio (PLR) = platelet count/lymphocyte count; monocyte-to-lymphocyte ratio (MLR) = monocyte count/lymphocyte count; and neutrophil-to-lymphocyte ratio (NLR) = neutrophil count/lymphocyte count.

### 2.5. Statistical Analysis

Statistical analyses were performed using Jamovi software, version 2.6.45 (The jamovi project, Sydney, Australia). The distribution of continuous variables was assessed using the Shapiro–Wilk test. Continuous variables were presented as mean ± standard deviation or median (interquartile range), as appropriate. Categorical variables were presented as numbers and percentages. Between-group comparisons were performed using the independent-samples t test or Mann–Whitney U test for continuous variables and the chi-square test or Fisher’s exact test for categorical variables, as appropriate. Receiver operating characteristic curve analysis was used to evaluate the ability of first-trimester composite indices to distinguish women who subsequently developed ICP from controls. Areas under the curve were reported with 95% confidence intervals, and optimal cutoff values were determined using the Youden index. Pairwise comparisons between the AUCs of the hepatobiliary indices were performed using DeLong’s test. Associations between the investigated indices and ICP were examined using univariable and multivariable binary logistic regression analyses and were reported as odds ratios with 95% confidence intervals. Because several hepatobiliary indices shared constituent laboratory parameters, the multivariable model was kept parsimonious to limit biochemical redundancy. The S-index was selected because it showed the highest observed discriminative performance, whereas the De Ritis ratio was selected as a complementary aminotransferase-based index that shares no constituent laboratory component with the S-index. GPR was not simultaneously included because it shares GGT and platelet count with the S-index, whereas TACPR incorporates AST and ALT, which also constitute the De Ritis ratio, as well as platelet count. Exclusion of these indices from the primary multivariable model was therefore intended to limit overlapping biochemical information rather than to imply inferior discriminative value. Multicollinearity among predictors in the multivariable models was assessed using variance inflation factors (VIFs) and tolerance values. Model 1 included the S-index and De Ritis ratio, whereas Model 2 additionally included maternal age, BMI and gestational age at blood sampling as clinically relevant covariates. Selected indices were rescaled to improve the interpretability of their odds ratios, with the corresponding increments reported in the table footnotes. Spearman correlation analysis was used to assess relationships between the indices and perinatal variables, and the results were visualized using a heatmap. All tests were two-sided, and a *p*-value <0.05 was considered statistically significant.

## 3. Results

A total of 293 pregnant women were included, comprising 96 women in the ICP group and 197 controls. Maternal age was comparable between the groups, whereas BMI was lower in the ICP group. First-trimester AST, ALT, and GGT levels were higher, whereas albumin and hemoglobin levels were lower in the ICP group. The S-index, De Ritis ratio, TACPR, GPR, and ALBI differed significantly between the groups, whereas SIRI, PLR, MLR, and NLR did not. Gestational age at delivery, birthweight, and 1- and 5-min Apgar scores were also lower in the ICP group (Table 1).

**Figure 1:**
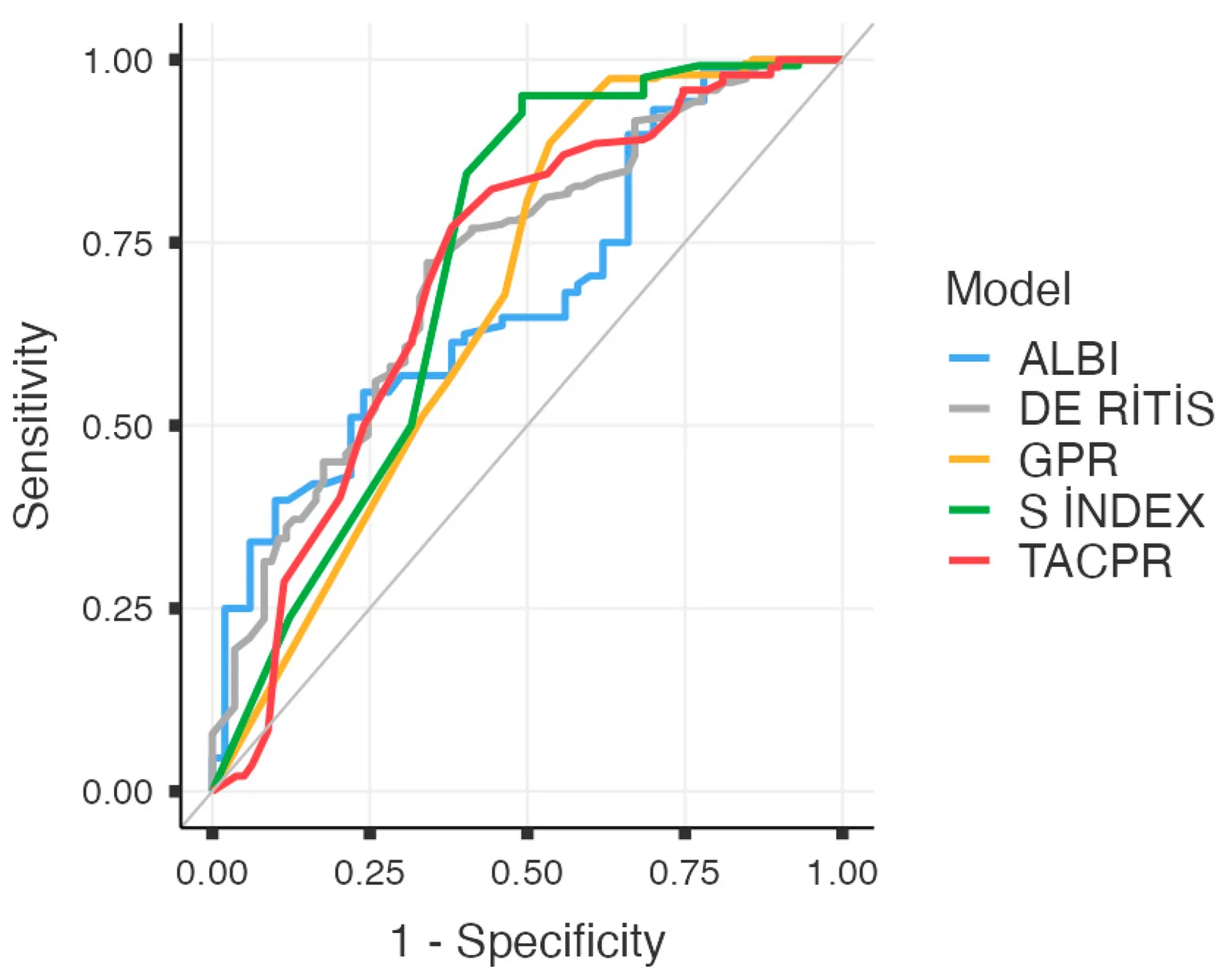
Receiver operating characteristic curves of first-trimester hepatobiliary indices for intrahepatic cholestasis of pregnancy.  ALBI, albumin–bilirubin score; GPR, gamma-glutamyl transferase-to-platelet ratio; TACPR, transaminase complex-to-platelet ratio. The grey diagonal line represents the reference line for no discrimination (AUC = 0.50).

The S-index had the highest observed AUC among the evaluated indices (0.808; 95% CI, 0.727–0.889). At a cutoff of ≥0.045, it yielded a sensitivity of 66.7% and a specificity of 95.1%. The De Ritis ratio, GPR, and TACPR also had AUCs significantly greater than 0.50, whereas the inflammatory indices showed no significant discrimination (Table 2, Figure 1 and Supplementary Figure S1). Pairwise DeLong comparisons showed no significant differences in AUC between the S-index and the De Ritis ratio, GPR, or TACPR; however, the AUC of the S-index was significantly higher than that of the ALBI (Supplementary Table S1).

In univariable analyses, the S-index, ALBI, GPR, and TACPR were positively associated with ICP, whereas the De Ritis ratio showed an inverse association. None of the inflammatory indices was significantly associated with ICP (Table 3). In the multivariable model adjusted for maternal age, BMI, and gestational age at blood sampling, the S-index and the De Ritis ratio remained significantly associated with ICP. A 0.01-unit increase in the S-index was associated with higher odds of ICP (aOR, 1.20; 95% CI, 1.06–1.35; *p* = 0.005), whereas a one-unit increase in the De Ritis ratio was associated with lower odds of ICP (aOR, 0.25; 95% CI, 0.09–0.67; *p* = 0.006) (Table 4).

No evidence of problematic multicollinearity was observed in the final multivariable model (maximum VIF, 1.06; minimum tolerance, 0.943); detailed model-fit and multicollinearity diagnostics are provided in Supplementary Table S2.

**Figure 2:**
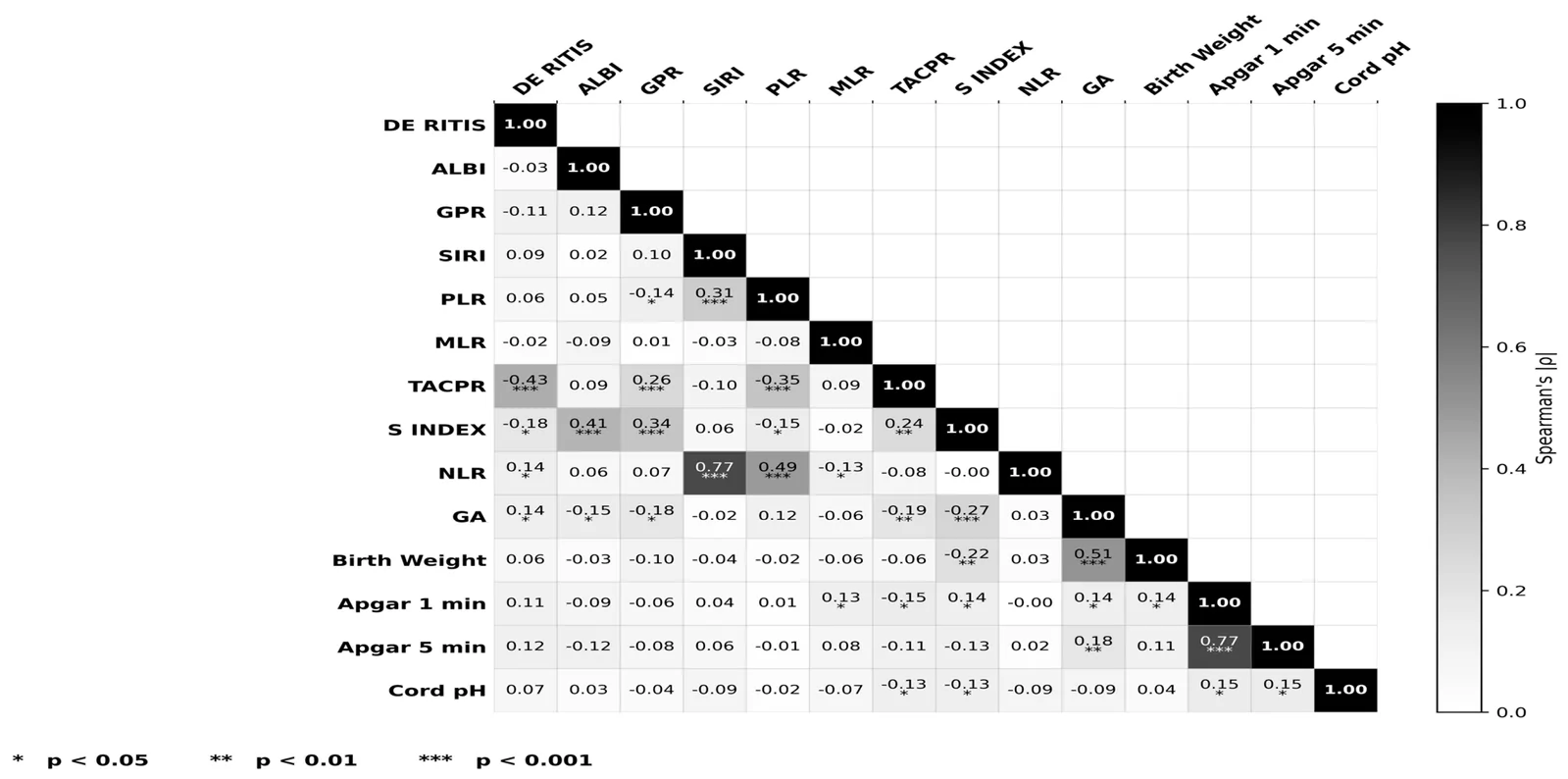
Spearman correlation matrix of first-trimester composite indices and perinatal outcomes. Spearman correlation coefficients (*ρ*) are displayed within the cells. Shading intensity represents the magnitude of the correlation (|*ρ*|), while the sign of the coefficient indicates the direction of the association. ALBI, albumin–bilirubin score; GPR, gamma-glutamyl transferase-to-platelet ratio; TACPR, transaminase complex-to-platelet ratio; SIRI, systemic inflammation response index; PLR, platelet-to-lymphocyte ratio; MLR, monocyte-to-lymphocyte ratio; NLR, neutrophil-to-lymphocyte ratio; GA, gestational age at delivery.

Correlations between the hepatobiliary indices and perinatal outcomes were generally weak. Among the evaluated indices, the S-index showed the largest correlations with gestational age at delivery and birthweight, although these associations remained weak in magnitude (Figure 2).

## 4. Discussion

In this retrospective case–control study, we systematically evaluated nine composite indices derived from routine first-trimester laboratory parameters in women who subsequently developed intrahepatic cholestasis of pregnancy. Hepatobiliary indices showed greater discriminative performance than inflammation-based indices. The S-index had the numerically highest observed AUC, followed by the De Ritis ratio, and both remained associated with ICP after adjustment for maternal age, BMI, and gestational age at blood sampling. These findings suggest that subtle alterations in hepatobiliary biochemical patterns may be detectable before the clinical manifestation of ICP, whereas no comparable pattern was detected using the evaluated leukocyte-derived inflammatory indices during the first trimester.

A biologically plausible explanation for this pattern is that ICP develops through the interaction between pre-existing hepatobiliary susceptibility and pregnancy-specific hormonal stress [4,5]. Variants in ABCB4, ABCB11, ATP8B1, and related pathways may reduce canalicular bile acid transport capacity [6], whereas increasing estrogen concentrations and sulfated progesterone metabolites later in gestation may further challenge this limited reserve and precipitate overt cholestasis [7]. Within this framework, the modest first-trimester differences in GGT, aminotransferase patterns, and albumin observed in our cohort should not be interpreted as early ICP but rather as a possible biochemical imprint of reduced hepatobiliary adaptability. Composite indices may make this subtle pattern more detectable by integrating complementary laboratory signals: the S-index combines GGT, albumin, and platelet count [15], whereas the De Ritis ratio reflects the relative distribution of aminotransferase activity [16]. The absence of a parallel pattern in the evaluated leukocyte-derived indices is also compatible with the possibility that systemic inflammatory changes emerge later or downstream of bile acid accumulation, hepatocellular stress, or placental involvement rather than representing the dominant early process [17,18]. Because transporter function, steroid metabolites, bile acid profiles, and inflammatory mediators were not directly measured, this interpretation remains hypothesis-generating.

Among the evaluated indices, the S-index emerged as the leading candidate marker, with the highest observed AUC of 0.808, and remained associated with ICP after multivariable adjustment. Originally developed as a noninvasive fibrosis score in chronic liver disease, the S-index integrates GGT, platelet count, and albumin within a single mathematical expression [15]. GGT reflects hepatobiliary enzyme activity, whereas albumin and platelet count contribute additional hepatic and hematological information to the index. Moreover, because albumin is squared in the denominator, even modest differences in albumin concentration may have a pronounced effect on the calculated value [15]. However, the individual components of the S-index were not directly compared with the composite index; therefore, whether the S-index provides incremental discriminative value beyond GGT, albumin, or platelet count remains uncertain.

The specificity of 95.1% observed at the Youden-derived cutoff suggests that an elevated first-trimester S-index identified a subgroup with a more pronounced hepatobiliary biochemical pattern within this cohort. Nevertheless, the observed sensitivity of 66.7% does not support its use as a stand-alone screening tool. Although the S-index significantly outperformed the ALBI, its AUC did not differ significantly from those of the De Ritis ratio, GPR, or TACPR. Furthermore, because the cutoff was derived and evaluated in the same cohort, its observed discriminative performance may be optimistic. Therefore, the S-index should be considered an exploratory marker associated with subsequent ICP and a potential candidate component of future prospectively validated multivariable risk-stratification models, rather than a stand-alone screening or diagnostic tool.

The De Ritis ratio also showed moderate discriminative performance for ICP, with an AUC of 0.717, and remained associated with ICP after adjustment for maternal age, BMI and gestational age at blood sampling (aOR, 0.25). Previous studies have similarly reported lower AST/ALT ratios in ICP, with proposed cutoffs ranging approximately from 1.1 to 1.3 [13,19]. In established ICP, ALT often increases more prominently than AST, resulting in a lower De Ritis ratio. Given that ALT is relatively more liver-specific, whereas AST is distributed across hepatic and extrahepatic tissues and has both cytosolic and mitochondrial forms, our findings may indicate that a subtle ALT-predominant hepatocellular pattern is already present during the first trimester in some women who subsequently develop ICP [16]. Thus, the De Ritis ratio may provide simple complementary information regarding the early hepatobiliary profile associated with subsequent ICP.

Among the remaining hepatobiliary indices, GPR and TACPR also demonstrated moderate discrimination, whereas ALBI showed lower discriminative performance. The absence of significant AUC differences between the S-index, GPR, and TACPR suggests that these indices may capture overlapping aspects of the same early hepatobiliary biochemical pattern. In contrast, SIRI, NLR, PLR, and MLR showed no meaningful first-trimester discrimination. This finding supports the possibility that systemic inflammatory changes become more apparent after bile acid accumulation or clinically established disease rather than during the preclinical phase [9,17,20]. However, the absence of significant findings for these indices does not exclude the involvement of inflammatory pathways that were not directly measured in the present study.

The potential clinical relevance of this study lies in the identification of an early hepatobiliary biochemical pattern associated with subsequent ICP using routinely available first-trimester laboratory data. The greater discriminative performance of hepatobiliary indices, particularly the high specificity observed for the S-index, supports further investigation of whether these markers can help define a subgroup requiring closer clinical observation. Following prospective validation, such markers might contribute to heightened awareness of subsequent pruritus and timely or repeated bile acid testing when clinically indicated. In this context, these indices could potentially lower the threshold for bile acid testing when suggestive symptoms develop and support closer follow-up of symptomatic women, particularly when initial bile acid concentrations are normal despite persistent symptoms. Earlier biochemical confirmation of ICP could, in turn, facilitate timely initiation of treatment, including ursodeoxycholic acid when clinically indicated, and allow subsequent obstetric management to be guided by established clinical criteria and bile acid concentrations. However, the present study did not evaluate whether their use shortens the time to diagnosis, changes clinical management, or improves maternal or perinatal outcomes. Given their moderate sensitivity and the case–control design, these indices should be regarded as candidate components of future multivariable risk-stratification models rather than stand-alone screening or diagnostic tools. Recent machine-learning models integrating clinical, biochemical, and hematological variables have shown promising performance for ICP prediction [21,22]. Whether such strategies lead to earlier diagnosis or improved maternal and perinatal outcomes requires prospective validation in unselected antenatal populations.

Maternal BMI was significantly lower in the ICP group than in controls. This finding is consistent with previous population-based evidence suggesting a nonlinear relationship between prepregnancy BMI and ICP risk [23]. However, BMI was not associated with ICP after adjustment in the multivariable model. The S-index and De Ritis ratio remained significantly associated with ICP after additional adjustment for maternal age, BMI, and gestational age at blood sampling. Nevertheless, prepregnancy BMI and BMI recorded at the first antenatal assessment may not be directly interchangeable, and this difference should be considered when comparing the present findings with previous studies.

The main strengths of this study are the use of laboratory measurements obtained before the clinical onset of ICP and the simultaneous comparison of nine hepatobiliary and inflammatory composite indices within the same first-trimester cohort. In addition, the evaluation of the less extensively studied S-index and the recently proposed TACPR alongside established indices provides comparative evidence regarding their potential roles in early ICP risk assessment. The contribution of the present study lies in the prediagnostic first-trimester assessment of two biologically distinct classes of composite markers within the same cohort, allowing direct comparison of their relative performance and comparative positioning of the less extensively studied S-index and recently proposed TACPR against established ICP-related indices.

Several limitations should be acknowledged. The retrospective, single-center case–control design limits the generalizability of the findings. Importantly, the control group did not represent an unselected population of asymptomatic pregnant women; controls were women who underwent fasting total bile acid testing because of clinical suspicion of ICP but were not diagnosed with ICP through delivery and had total bile acid concentrations <10 µmol/L at all available measurements. This clinically selected control group may have introduced selection and spectrum bias and limits the generalizability of the observed associations and discriminatory performance to an unselected obstetric population. Because case–control sampling does not reflect the population prevalence of ICP, positive and negative predictive values, absolute risk, and model calibration could not be estimated. Accordingly, the observed associations and AUC estimates should not be interpreted as prospective predictive performance or screening accuracy in an unselected obstetric population. The ROC cutoff values were derived and evaluated within the same cohort, without internal resampling or external validation; therefore, both the selected thresholds and their observed discriminatory performance may be optimistic and should be considered exploratory until independently validated. Several indices were originally developed in populations with chronic liver disease or hepatocellular carcinoma, and pregnancy-specific reference ranges and clinically validated thresholds have not been established. In addition, the individual biochemical components (e.g., GGT, AST, ALT, albumin, and platelet count) were not directly compared with their corresponding composite indices; therefore, the incremental discriminative value of the composite calculations beyond routine laboratory parameters remains uncertain. The indices were calculated from a single first-trimester assessment, while shared biochemical components limited their simultaneous inclusion in multivariable models. Consequently, the exclusion of some hepatobiliary indices from the final model should not be interpreted as evidence that they lack independent value. Although gestational age at first-trimester blood sampling was additionally included in the revised multivariable model together with maternal age and BMI, residual confounding remains possible. Owing to the retrospective design, several potentially relevant variables, including smoking status, previous ICP, family history of ICP, seasonality, and transporter gene variants, were not consistently available and could not be evaluated as potential confounders. Peak bile acid concentrations, disease severity, treatment, and indications for delivery were not incorporated into outcome-specific models, precluding assessment of whether these indices were independently associated with perinatal outcomes. Finally, the long study period may have encompassed changes in laboratory methods and clinical management. Prospective multicenter studies incorporating serial measurements, internal and external validation, and direct comparison with individual laboratory components are therefore required before clinical application. Cord blood pH was unavailable for a substantial proportion of participants, particularly in the ICP group; therefore, the absence of a significant between-group difference in cord pH should be interpreted cautiously.

## 5. Conclusions

First-trimester hepatobiliary composite indices were associated with subsequent ICP, with the S-index and De Ritis ratio remaining significant after adjustment for maternal age, BMI, and gestational age at blood sampling. The S-index showed the numerically highest observed AUC, although its discriminative performance was not statistically different from that of the De Ritis ratio, GPR, or TACPR. These findings suggest the presence of a detectable hepatobiliary biochemical pattern preceding the clinical onset of ICP, whereas no comparable pattern was identified using the inflammatory indices evaluated during the first trimester. Because these indices can be calculated from routine antenatal laboratory parameters, they may have potential as adjunctive markers in future ICP risk-stratification strategies. However, the present study did not establish whether their use improves symptom surveillance, shortens the interval to biochemical confirmation, or changes clinical management. Prospective multicenter studies incorporating serial measurements and internal and external validation are needed to establish their clinical utility and determine whether their integration into antenatal care improves maternal or perinatal outcomes.

## Figures and Tables

**Table 1:** Maternal, first-trimester laboratory, composite indices and perinatal characteristics of the study population.

Variable	Control (*n* = 197)	ICP (*n* = 96)	*p* Value
Maternal characteristics			
Maternal age (years)	28.9 ± 5.5	29.9 ± 6.5	0.147
BMI (kg/m^2^)	30.3 ± 5.0	26.6 ± 7.3	<0.001
Gestational age at blood sampling (weeks)	10.0 (3.4)	10.1 (3.1)	0.407
First-trimester laboratory parameters			
AST (U/L)	15 (5)	18 (12)	<0.001
ALT (U/L)	12 (7)	18 (17.3)	<0.001
GGT (U/L)	7 (5)	17 (20.8)	<0.001
Total bilirubin (µmol/L)	3.14 (5.13)	3.56 (6.33)	0.161
Albumin (g/L)	41.9 ± 5.9	38.1 ± 9.1	0.002
Hemoglobin (g/dL)	12.5 ± 2.9	11.8 ± 1.3	0.006
Platelet count (×10^3^/µL)	266 ± 67.5	259 ± 66.9	0.530
Neutrophil count (×10^3^/µL)	6.28 ± 1.88	6.48 ± 2.51	0.889
Lymphocyte count (×10^3^/µL)	1.97 ± 0.60	1.99 ± 0.66	0.766
Monocyte count (×10^3^/µL)	0.567 ± 1.13	0.563 ± 0.44	0.162
Hepatobiliary composite indices			
De Ritis ratio	1.23 (0.64)	0.90 (0.53)	<0.001
ALBI	−3.05 (0.75)	−2.75 (0.61)	<0.001
GPR	0.043 (0.030)	0.065 (0.090)	<0.001
TACPR	0.659 (0.560)	1.429 (2.158)	<0.001
S-index	0.015 (0.010)	0.060 (0.080)	<0.001
Inflammatory composite indices			
SIRI	1.46 (0.97)	1.50 (1.07)	0.465
PLR	131 (55.8)	132 (46.7)	0.286
MLR	0.240 (0.110)	0.250 (0.120)	0.387
NLR	3.09 (1.67)	3.43 (1.85)	0.754
Perinatal outcomes			
Gestational age at delivery (weeks)	38.9 ± 1.5	36.8 ± 1.7	<0.001
Birth weight (g)	3298 ± 470	2851 ± 708	<0.001
Apgar score at 1 min	8 (2)	7 (1)	0.004
Apgar score at 5 min	10 (1)	9 (1.75)	0.001
Cord blood pH †	7.31 ± 0.058	7.31 ± 0.074	0.542

Data are presented as mean ± standard deviation (SD) or median (interquartile range [IQR]), as appropriate. Normality of continuous variables was assessed using the Shapiro–Wilk test. Between-group comparisons were performed using the independent-samples *t* test or the Mann–Whitney U test, as appropriate. † Cord blood pH was available for 63/96 (65.6%) women in the ICP group and 165/197 (83.8%) women in the control group. Because umbilical cord blood gas analysis was not routinely performed in all newborns, cord pH was analyzed using available cases only. ICP, intrahepatic cholestasis of pregnancy; BMI, body mass index; AST, aspartate aminotransferase; ALT, alanine aminotransferase; GGT, gamma-glutamyl transferase; ALBI, albumin–bilirubin score; GPR, gamma-glutamyl transferase-to-platelet ratio; TACPR, transaminase complex-to-platelet ratio; SIRI, systemic inflammation response index; PLR, platelet-to-lymphocyte ratio; MLR, monocyte-to-lymphocyte ratio; NLR, neutrophil-to-lymphocyte ratio.

**Table 2:** Receiver operating characteristic analysis of first-trimester composite indices for intrahepatic cholestasis of pregnancy.

Biomarker	AUC (95% CI)	*p* Value	Cut-Off	Sensitivity (%)	Specificity (%)
S Index	0.808 (0.727–0.889)	<0.001	≥0.045	66.67	95.08
De Ritis ratio	0.717 (0.651–0.782)	<0.001	≤1.045	65.88	72.25
GPR	0.713 (0.638–0.788)	<0.001	≥0.055	53.57	95.34
TACPR	0.706 (0.632–0.780)	<0.001	≥0.135	62.03	77.08
ALBI	0.680 (0.589–0.770)	<0.001	≥−2.945	76.00	54.55
MLR	0.533 (0.459–0.607)	0.386	≥0.235	61.90	48.17
SIRI	0.528 (0.452–0.604)	0.474	≥1.915	40.24	71.20
NLR	0.512 (0.435–0.589)	0.762	≥3.405	52.38	59.69
PLR	0.458 (0.383–0.533)	0.274	≥131.505	52.63	51.58

Receiver operating characteristic (ROC) curve analysis was performed to assess the ability of the investigated indices to distinguish women who developed ICP from controls. Optimal cutoff values were determined using the Youden index. AUCs are reported with 95% confidence intervals, whereas sensitivity and specificity are presented as percentages. ICP, intrahepatic cholestasis of pregnancy; AUC, area under the curve; CI, confidence interval; ALBI, albumin–bilirubin score; GPR, gamma-glutamyl transferase-to-platelet ratio; TACPR, transaminase-to-platelet ratio; MLR, monocyte-to-lymphocyte ratio; SIRI, systemic inflammation response index; NLR, neutrophil-to-lymphocyte ratio; PLR, platelet-to-lymphocyte ratio.

**Table 3:** Univariable logistic regression analyses of the associations between first-trimester composite indices and intrahepatic cholestasis of pregnancy.

Predictor	OR (95% CI)	*p* Value
S-index	1.35 (1.20–1.52)	<0.001
De Ritis ratio	0.15 (0.07–0.30)	<0.001
ALBI	2.88 (1.52–5.47)	0.001
GPR	1.26 (1.17–1.36)	<0.001
TACPR	2.06 (1.42–2.98)	<0.001
SIRI	1.07 (0.95–1.19)	0.271
PLR	0.996 (0.992–1.000)	0.134
MLR	0.94 (0.73–1.21)	0.633
NLR	1.00 (0.93–1.08)	0.955

Odds ratios for GPR and the S-index are reported per 0.01-unit increase in the original scale to improve interpretability, whereas odds ratios for all other variables correspond to a one-unit increase in their original scales. Rescaling does not alter p values, statistical significance, or model fit. OR, odds ratio; CI, confidence interval; ALBI, albumin–bilirubin score; GPR, gamma-glutamyl transferase-to-platelet ratio; TACPR, transaminase complex-to-platelet ratio; SIRI, systemic inflammation index; PLR, platelet-to-lymphocyte ratio; MLR, monocyte-to-lymphocyte ratio; NLR, neutrophil-to-lymphocyte ratio.

**Table 4:** Multivariable logistic regression models for intrahepatic cholestasis of pregnancy.

Variable	Model 1 OR (95% CI)	*p*	Model 2 OR (95% CI)	*p*
S-index	1.27 (1.13–1.43)	<0.001	1.20 (1.06–1.35)	0.005
De Ritis ratio	0.23 (0.09–0.58)	0.002	0.25 (0.09–0.67)	0.006
Maternal age	—	—	0.98 (0.93–1.06)	0.549
BMI	—	—	0.93 (0.85–1.02)	0.118
Gestational age at blood sampling (weeks)			1.10 (0.88–1.37)	0.411

Model 1 included the S-index and De Ritis ratio, whereas Model 2 additionally included maternal age, BMI, and gestational age at blood sampling. Results are presented as odds ratios (ORs) with 95% confidence intervals (CIs). For interpretability, the odds ratio for the S-index is reported per 0.01-unit increase in its original scale. Odds ratios for all other continuous variables correspond to a one-unit increase in their original scales. BMI, body mass index; OR, odds ratio; CI, confidence interval.

## Data Availability

The data presented in this study are available from the corresponding author upon reasonable request. The data are not publicly available because of institutional and ethical restrictions related to the protection of patient confidentiality.

## Supplementary Materials

The following supporting information can be downloaded at https://www.mdpi.com/article/10.3390/diagnostics16172745/s1: Table S1: Pairwise comparison of ROC curve areas for first-trimester hepatobiliary indices using DeLong’s test; Table S2: Model fit and multicollinearity diagnostics for the multivariable logistic regression models; Figure S1: Receiver operating characteristic curves of first-trimester inflammatory composite indices for intrahepatic cholestasis of pregnancy.
