# Supplementary material for: First-Trimester Hepatobiliary and Inflammatory Composite Indices as Candidate Markers for Subsequent Intrahepatic Cholestasis of Pregnancy: A Case–Control Study
Source: Diagnostics (Basel). 2026 Aug 27;16(17):2745. doi: 10.3390/diagnostics16172745 (PMC13564545; doi:10.3390/diagnostics16172745)
Supplement: Supplementary file 1 [file diagnostics-16-02745-s001.zip › diagnostics-4483051-supplementary.pdf]

Supplementary Table S1. Pairwise comparison of ROC curve areas for first-trimester hepatobiliary indices using DeLong's test

**Comparison**	**ΔAUC**	**p value**
S-index vs. De Ritis ratio	0.092	0.214
S-index vs. GPR	0.095	0.215
S-index vs. TACPR	0.102	0.211
S-index vs. ALBI score	0.129	<0.001
De Ritis ratio vs. ALBI score	0.037	0.302
De Ritis ratio vs. GPR	0.004	0.835
De Ritis ratio vs. TACPR	0.010	0.899
GPR vs. ALBI score	0.033	0.005
GPR vs. TACPR	0.007	0.837
TACPR vs. ALBI score	0.027	0.452

Data represent pairwise comparisons of the areas under the receiver operating characteristic curves using DeLong's test. ΔAUC was calculated as the AUC of the first-listed index minus the AUC of the second-listed index; therefore, a positive value favors the first-listed index. All p values are two-sided. AUC, area under the curve; ROC, receiver operating characteristic; ALBI, albumin–bilirubin score; GPR, gamma-glutamyl transferase-to-platelet ratio; TACPR, transaminase complex-to-platelet ratio.

Supplementary Table S2. Model fit and multicollinearity diagnostics for the multivariable logistic regression models

**Model characteristic**	**Model 1: Hepatobiliary model**	**Model 2: Final model**
**Predictors**	**De Ritis ratio, S Index**	**Age, BMI, De Ritis ratio, S-Index, Gestational age at blood sampling (weeks)**
**AIC**	163	143
**McFadden R^2**	0.260	0.274
**Nagelkerke R^2**	0.388	0.398
**AUC**	0.836	0.846
**Maximum VIF**	1.02	1.06
**Minimum tolerance**	0.983	0.943

AIC, Akaike information criterion; AUC, area under the receiver operating characteristic curve; VIF, variance inflation factor.

Supplementary Figure S1. Receiver operating characteristic curves of first-trimester inflammatory composite indices for intrahepatic cholestasis of pregnancy

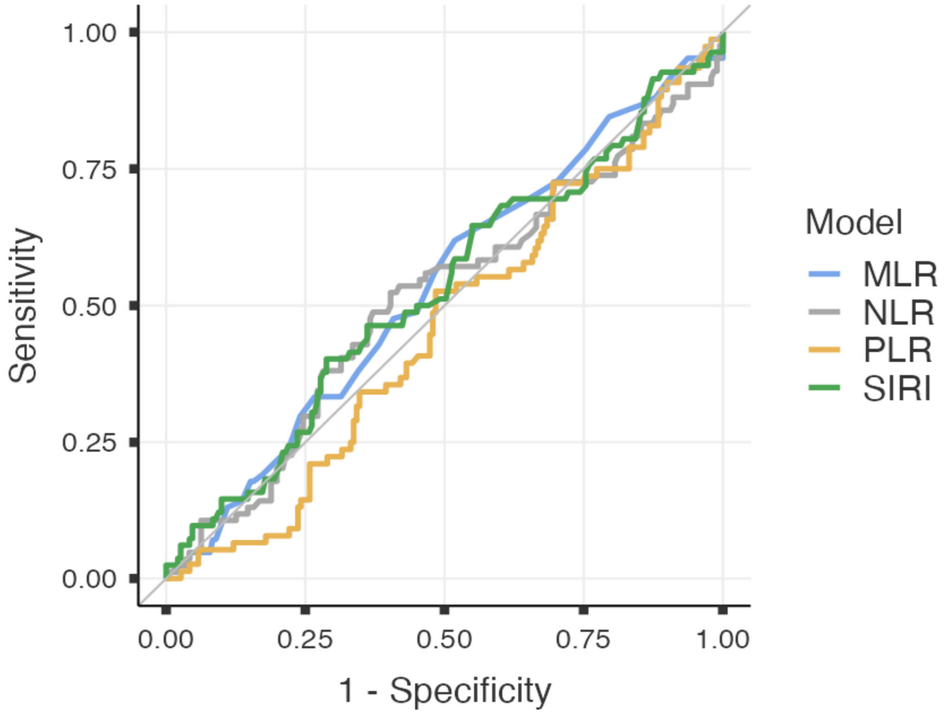

ROC, receiver operating characteristic; ICP, intrahepatic cholestasis of pregnancy; SIRI, systemic inflammation response index; PLR, platelet-to-lymphocyte ratio; MLR, monocyte-to-lymphocyte ratio; NLR, neutrophil-to-lymphocyte ratio.
